# The Intrinsic Estimator, Alternative Estimates, and Predictions of Mortality Trends: A Comment on Masters, Hummer, Powers, Beck, Lin, and Finch

**DOI:** 10.1007/s13524-016-0476-8

**Published:** 2016-05-12

**Authors:** Manfred te Grotenhuis, Ben Pelzer, Liying Luo, Alexander W. Schmidt-Catran

**Affiliations:** 1 Department of Sociology and Social Sciences Research Methods, Radboud University, P.O. Box 9104, 6500 HE Nijmegen, The Netherlands; 2 Department of Sociology and Criminal Justice, University of Delaware, Newark, DE USA; 3 Institute of Sociology and Social Psychology, University of Cologne, Cologne, Germany

## Abstract

In this article, we discuss a study by Masters et al. (2014), published in *Demography*. Masters and associates estimated age, period, and cohort (APC) effects on U.S. mortality rates between 1959 and 2009 using the intrinsic estimator (IE). We first argue that before applying the IE, a grounded theoretical justification is needed for its fundamental constraint on minimum variance of the estimates. We next demonstrate IE’s high sensitivity to the type of dummy parameterization used to obtain the estimates. Finally, we discuss challenges in the interpretation of APC models. Our comments are not restricted to the article in question but pertain generally to any research that uses the IE.

## Introduction

In their article “Long-Term Trends in Adult Mortality for U.S. Blacks and Whites: An Examination of Period- and Cohort-Based Changes,” Masters et al. (2014) used the intrinsic estimator (IE) to estimate age, period, and cohort (APC) effects on mortality rates for whites and blacks in the United States. While concerns have been raised about the use of the IE technique (Fienberg 2013; Luo 2013a; O’Brien 2011), Masters et al. (2014:2066) argued that the IE is the “preferred solution” to find the true APC effects on mortality. However, as Luo (2013a) demonstrated, without external information, we do not know whether the IE recovers the parameters that generated the data. In addition, acknowledging that the IE is sensitive to the choice of the reference—or, more accurately, omitted—categories for each age, period, and cohort effect, Masters et al. found their results highly consistent using different omitted categories (Masters et al. 2014: Online Resource 1). However, these sensitivity analyses tend to be consistent as long the same parameterization framework is used (Luo et al. forthcoming; Pelzer et al. 2015).

Our comments on the Masters et al. (2014) article are threefold. First, the IE estimates in their article do not necessarily recover the parameters that generated these mortality data; that is, the estimates could very well be biased. Second, Masters and associates provided limited sensitivity analysis. We will show that the IE estimates can change dramatically with different choices of omitted categories of age, period, and cohort. Third, because of the perfect dependency between age, period, and cohort, we raise concerns about the meaning and interpretation of the estimated mortality rates.

## Theoretical Basis for the Intrinsic Estimator’s Fundamental Assumption

The literature warns researchers that the identification problem (i.e., age = period – birth cohort) is inherent in APC models and that no technical solution overcomes this problem (Bell and Jones 2015; Fienberg 2013; Fienberg et al. 2015; Held and Riebler 2013; Luo 2013a, b; Luo et al. forthcoming; Winship and Harding 2008). Therefore, researchers may need to choose an estimator based on theoretical grounds. Without such theoretical justification, it remains questionable whether the estimates obtained are unbiased and consistent of the true age, period, and cohort parameters that generated the data.

The IE estimator circumvents the identification problem by using a specific constraint on the estimates for age, period, and cohort. Using the method provided by Kupper et al. (1985) and Luo (2013a), the specific form of the constraint that the IE uses can be computed and written as follows (using three age groups, three periods, and five cohorts):

1 $$ \left(1,-1,2,1,-1\right)\left(\begin{array}{c}\hfill {\upbeta}_1^A\hfill \\ {}\hfill {\upbeta}_1^P\hfill \\ {}\hfill {\upbeta}_1^C\hfill \\ {}\hfill {\upbeta}_2^C\hfill \\ {}\hfill {\upbeta}_4^C\hfill \end{array}\right)=0. $$

The first (row) vector in Eq. (1) is called the *null vector* (**b**_0_), and its numbers are solely a function of the number of age, period, and cohort categories in a given data set. The second (column) vector (**b**_*IE*_) holds the IE estimates. The IE constraint is that the multiplication of both vectors equals zero.

**Fig. 1:**
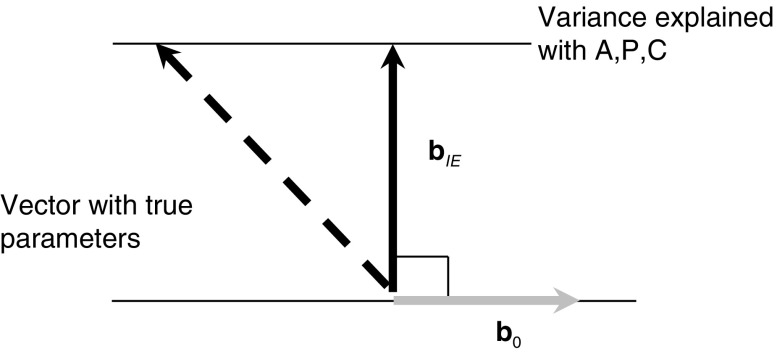
The IE constraint, with **b**_0_ (grey line), **b**_*IE*_ (solid line), and a possible vector with the set of parameters that generated the data (dashed line)

Figure 1 represents the IE constraint and its components. The vector with the IE estimates (**b**_*IE*_)—set perpendicular to **b**_0_—is the shortest route from the origin to the horizontal line that represents the variance explained by all age, period, and cohort groups. Because the vector is shortest, the IE estimates have the smallest variance; that is, the sum of squared IE estimates is smallest. Any other set of estimates with exactly the same explanatory power will have a larger variance in its estimates. Whether the parameters that generated the data also have smallest variance, however, is unknown. Therefore, the IE estimates can be severely biased, as depicted in Fig. 1, in which the true parameter vector is very different from the IE estimates vector.

Thus, the IE estimates obtained by Masters and colleagues are valid if and only if the underlying data-generating mechanism holds parameters for age, period, and cohort that also have the lowest variance possible. If, however, the parameters do not follow this constraint (see the dashed line in Fig. 1), then the IE estimates (the solid line in Fig. 1) are biased. Masters and associates provide little external information or theoretical consideration to verify the validity of the IE constraint of minimum variance in Eq. (1) and shown in Fig. 1.

Masters and colleagues contend that the IE can be applied in their research because the mortality data they use contain nonlinear effects, whereas “Luo set the functional forms of all APC effects on the outcome Y to be exactly linear” (2014: Online Resource 1). However, Luo (2013a) demonstrated that the IE estimates are also invalid when the IE’s constraint is not satisfied in simulated data with both linear and nonlinear effects (see, e.g., Luo 2013a: figure 1, 1963–1964). The IE produces unbiased and consistent estimates only when the minimum variance constraint also applies to the data generating parameters. This is equally true when the age, period, and cohort effects all are perfectly linear (see, e.g., Data Set 1 in Luo 2013a: table 3, 1954).

Masters and colleagues performed some interesting simulation analyses and argued that the IE can recover the “true” age, period, and cohort effects specified in the simulation function (Masters et al. 2014: figure S3, Online Source 1). However, the IE performed well in their simulations simply because all of their simulation functions satisfy the IE’s constraint of minimum variance. In other words, it is their method of simulating the data, not the statistical properties of the IE, that guarantees the IE’s performance.

Specifically, Masters and associates specified the data-generating mechanism “us[ing] the estimated coefficients from IE models,” and then “refit the IE estimator models to the simulated data to determine how well the IE can retrieve the original effects in the presence of the simulated error” (Masters et al. 2014: Online Resource 1). However, the numeric values of the IE estimates are used in the function that generates the simulated data. As a result, the simulated vectors with IE estimates all have close to minimum variance and therefore, as we have already discussed, the IE produces a set of estimates on the grounds that Eq. (1) is correct—that is, the IE’s constraint is satisfied by the data-generating mechanism. Using the numeric values of these IE estimates in the simulation function means that for the resulting simulated data, the IE constraint in Eq. (1) is at least approximately satisfied. Because the IE is an unbiased estimator when its constraint conforms to the truth (Luo 2013a:1958), the IE will appear to yield reliable estimates as it did in Masters et al. (2014: figure S3, Online Resource 1).

This simulation procedure is therefore not very useful to demonstrate the unbiasedness of the IE estimates because any APC estimator can recover the age, period, and cohort parameters when the data are generated with the constraint applied by that estimator, as O’Brien (2011:436) noted. For example, one may model the same mortality data using the constrained generalized linear model (CGLM; Mason et al. 1973) while setting the parameters of the first two age groups as equal. The set of age, period, and cohort effects obtained this way are then used in the data-generating function while adding some random error. Finally, one reestimates the model using the CGLM constraint on the first two age groups to find that the CGLM recovered the parameters that were used to simulate the data set. Of course, the CGLM estimates are only reliable in this example because the data were generated to satisfy its constraint in the first place (i.e., to have equal effects for the first two age groups). In fact, an infinite number of possible solutions exist that can produce the same mortality data with some random error, and the IE and the CGLM are just two of them. If the data-generating mechanism satisfies the IE constraint, as in the simulation in Master et al.’s article, then the IE can yield valid estimates; otherwise, the IE can be wildly biased. We elaborate on this crucial argument against the use of the IE in the next section.

## A Matter of Choice: IE’s Dependence on Parameterization

Masters et al. obtained their IE estimates using a dummy parameterization (see their table S4 in Online Resource 1) known as *effect coding* (Hardy 1993). This coding scheme results in estimates for all age, period, and cohort dummy variables that are deviations from the scaled grand mean. The choice to use effect coding should be arbitrary because under normal conditions, the way of coding the dummy variables is of no real importance. For instance, in dummy coding, estimates are scaled as deviations from the reference category, and they differ from effect-coded estimates only by a scaling factor. However, because the effect coding is part of the constraint (of minimum variance) in the IE to solve the APC identification problem, the IE estimates obtained from effect coding and the IE estimates from dummy coding can differ substantially from each other (Luo et al. forthcoming; Pelzer et al. 2015).

**Fig. 2:**
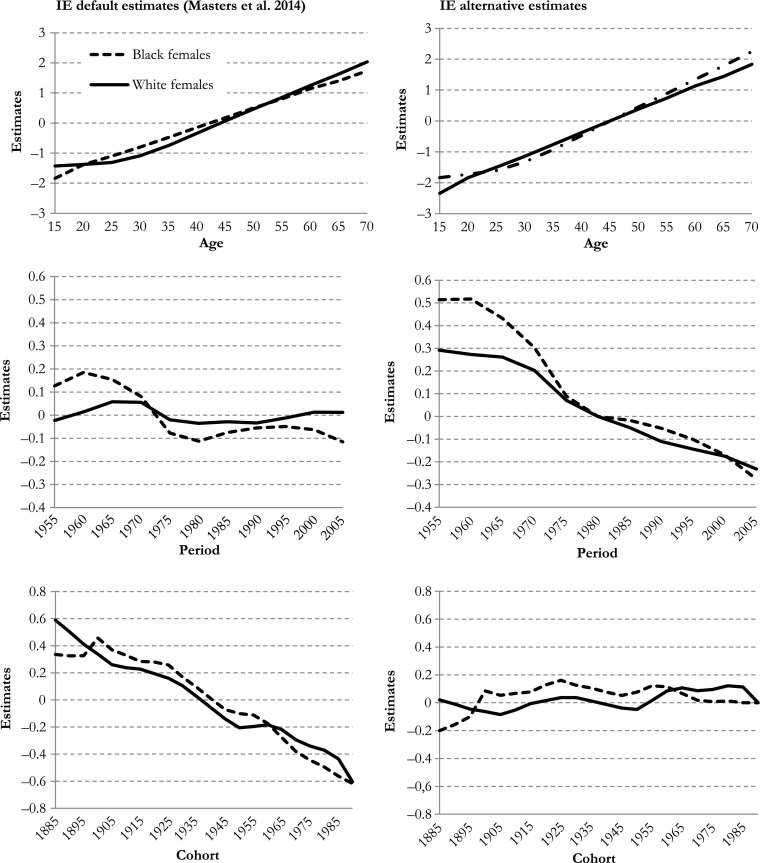
IE default (Masters et al. 2014) with effect coding and IE alternative estimates for all all-cause mortality in black and white females using dummy coding

Masters and associates examined the robustness of the IE results by using different sets of omitted categories, but all of them remain *within* the same parameterization framework (i.e., effect coding). As Pelzer et al. (2015) showed, the outcomes then are rather stable, especially when the data contain a large set of different age, period, and cohort groups, like in this case. The more comprehensive and helpful examination is to change the parameterization framework from effect coding to dummy coding and compare the IE estimates under these different parameterizations. Figure 2 illustrates how the IE estimates of the age, period, and cohort effects change with the choice of parameterization. The left panels of Fig. 2 present the original IE estimates in Masters et al.’s article obtained with effect coding, and the right panels show an alternative set of IE estimates with dummy coding (omitted are the middle categories of age (45–49) and period (1980–1984), and the last category of cohort (1990–1995)). Although both the IE with effect coding and the IE with dummy coding lead to exactly the same predicted mortality rates, they differ in estimated effects. The period and cohort differences are striking: whereas Masters et al.’s estimates suggest a strong negative trend in cohort effects and no clear trend in period effects, our estimates suggest limited cohort differences but a strong negative trend in period effects.

Pelzer et al. (2015) demonstrated that each parameterization framework relates to a particular IE constraint, which causes the estimates to be fundamentally different (see Fig. 2). In the default IE that Masters and associates used, the effect coding framework is part of the constraint: the estimates obtained this way have the lowest variation possible. That is, the variance of the set of IE estimates is kept at its minimum (see Fig. 1). In this sense, the IE is a member of a larger family of constrained models that have been developed in the past, and the main criticism of those models applies here as well: verifying that the true parameters for age, period, and cohort in the data generating process also have minimum variation requires external information to determine which particular framework is the one that generated the data—a task that may be impossible. It is therefore questionable whether the default IE estimates presented by Masters et al. (2014) are unbiased: do they reflect the unknown parameters or not?

## Pitfalls in Interpreting APC Estimates in Regression Models

A less-discussed aspect of APC models is the potential difficulty in interpreting the resulting estimates. In regression models, the effect of a predictor variable is typically interpreted as the pure or independent effect of that predictor when keeping the other predictors constant. However, this type of interpretation makes less sense in APC models, in which one of the predictors (e.g., birth cohort) does not vary when the other two (age and period) are kept constant. Consequently, it is unfeasible to demonstrate change in the dependent variable by manipulating the value of only one predictor.

**Fig. 3:**
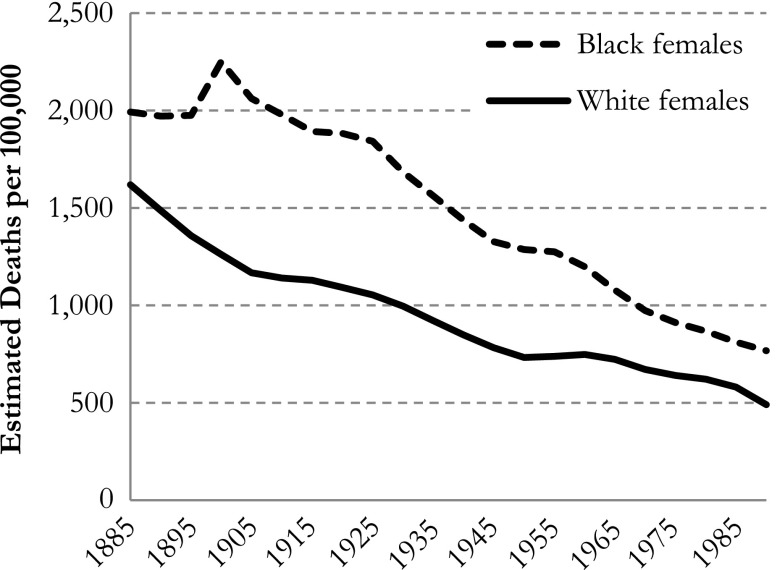
IE default (Masters et al. 2014: figure 1) estimated all-cause mortality rates in black and white females, for cohort (at ages 60–64, and period 1975–1979)

For example, Masters et al. (2014) showed the temporal trends in estimated mortality rates across birth cohorts 1885–1990 for age 60–64 and period 1975–1979. Obviously, in that combination of period and age group, one can observe only the cohort group 1910–1919 and not the entire birth range of 1885–1990. For ease of comparison, in Fig. 3 we display part of Masters et al.’s first figure (p. 2058).

**Fig. 4:**
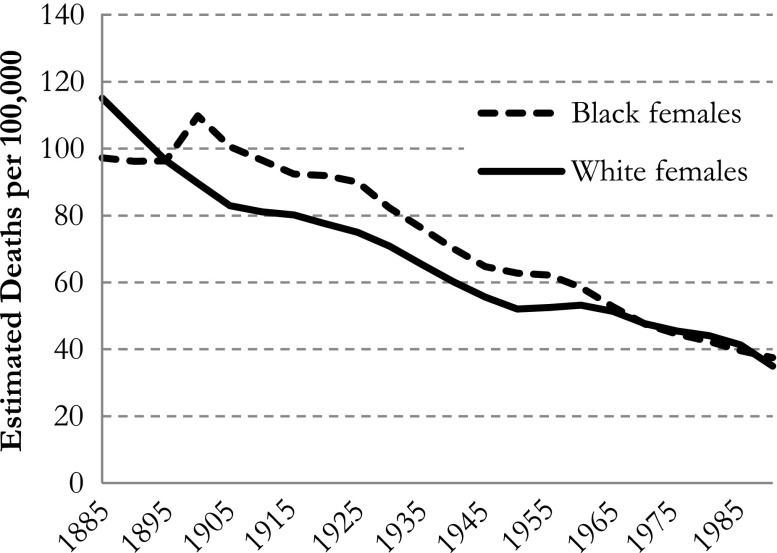
IE default (Masters et al. 2014) estimated all-cause mortality rates in black and white females, for cohort (at ages 15–19 and period 2005–2009). Note the different scaling of the *y*-axes compared with Figs. 3 and 5

Furthermore, in the APC regression models with dummy variables, the predicted mortality rate in each cohort depends on which age group and period is chosen. So, comparing cohort trends for blacks and whites by referring to one particular age group and period may lead to conclusions that hold for that specific cohort and period combination but generally not for other cohorts and/or periods. To illustrate our point, we calculated the estimated mortality rates for all cohorts using the first age group and the last period as a baseline (see Fig. 4).

**Fig. 5:**
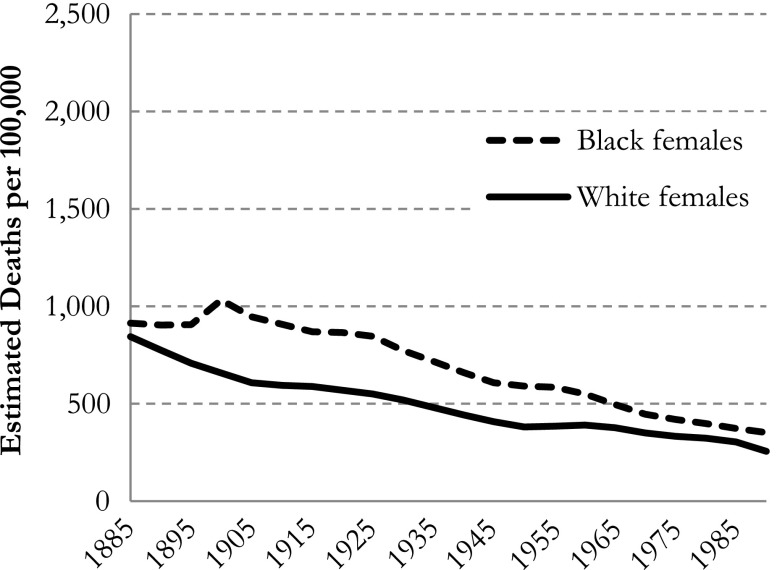
IE default (Masters et al. 2014) estimated all-cause mortality rates in black and white females, for cohort as deviations from the sample mortality rate in blacks and whites, respectively. Scaling of *y*-axis is equal to that of Fig. 3

As shown in Fig. 4, the mortality rates are much lower now (as to be expected given the young age group). More important, however, we find lower mortality rates for black females than for white females in the first cohorts but almost equal rates for black females and white females in the last cohorts—results that are rather different from Masters et al.’s (2014) results (see our Fig. 4 vs. Fig. 3). To avoid these problems in comparing whites’ and blacks’ mortality rates, one may present predictions without using an arbitrarily chosen age group, period, or cohort. Instead, the average mortality rate (total number of deaths divided by the total number of exposures) can be used as a reference (see Fig. 5).

In Fig. 5, the sample mean of the estimated mortality rates is equal to the overall mortality rate for blacks and whites, respectively. This way, the comparison of the trends between blacks and whites no longer depends on which categories are chosen. Compared with the results in Masters et al. (2014: figure 3), our results tell a different story about differences between blacks and whites, which underpins our concerns about presenting estimated mortality rates with fixed data points.
